# Association Between Interictal Sensory Hypersensitivities and Vestibular Symptoms in Migraine: A Cross‐Sectional Study

**DOI:** 10.1002/brb3.70874

**Published:** 2025-10-20

**Authors:** Alex Jaimes, Jaime Rodríguez‐Vico, Olga Pajares, Andrea Gómez, Jesús Porta‐Etessam

**Affiliations:** ^1^ School of Medicine Autonomous University of Madrid Madrid Spain; ^2^ Headache Unit, Neurology Department Fundación Jiménez Díaz University Hospital Madrid Spain

**Keywords:** dizziness, osmophobia, phonophobia, photophobia, vertigo

## Abstract

**Background:**

Dizziness and vertigo are common in migraine patients. This study investigated the relationship between vestibular symptoms and interictal hypersensitivities.

**Methods:**

A cross‐sectional survey was conducted in migraine patients from a headache unit and a neurology clinic.

**Results:**

Among 274 participants (87.2% female, mean age 41.7 ± 12.4 years), interictal sensory hypersensitivities were common: 76 (27.7%) reported photophobia, 67 (24.5%) phonophobia, and 69 (25.2%) osmophobia. Vestibular symptoms were also common: dizziness 148 (54.0%) and vertigo 119 (43.8%).Vestibular symptoms were more prevalent in patients with interictal symptoms: photophobia (dizziness 77.6% vs. 47.1%, *p* < 0.001; vertigo 68.0% vs. 33.8%, *p* < 0.001), phonophobia (dizziness 74.6% vs. 47.3%, *p* < 0.001; vertigo 61.2% vs. 37.7%, *p* < 0.001), and osmophobia (dizziness 66.7% vs. 49.8%, *p* = 0.015; vertigo 56.5% vs. 39.4%, *p* = 0.011). Patients with interictal sensory hypersensitivities had higher Dizziness Handicap Inventory (DHI) scores: photophobia (57.9 ± 20.7 vs. 45.1 ± 22.3, *p* < 0.001), phonophobia (55.6 ± 20.2 vs. 47.5 ± 23.2, *p* = 0.037), and osmophobia (53.1 ± 22.3 vs. 48.9 ± 22.5, *p* = 0.299).In multivariable analysis, the number of interictal hypersensitivities predicted dizziness (OR 1.74, *p* = 0.002) and vertigo (OR 1.72, *p* < 0.001). Having ≥ 15 headache days/month was the strongest predictor of dizziness (OR 3.30, *p* = 0.001), while nausea was the strongest predictor of vertigo (OR 3.86, *p* < 0.001).

**Conclusions:**

Vestibular symptoms are highly prevalent in migraine and strongly associated with interictal hypersensitivities. The number of affected sensory modalities predicts both prevalence and severity. Higher burdens of migraine and nausea are key predictors of dizziness and vertigo, respectively.

## Graphical abstract

This cross‐sectional study of 274 migraine patients examined the association between interictal hypersensitivities (photophobia, phonophobia, and osmophobia) and vestibular symptoms. Vestibular symptoms were significantly more frequent in patients with interictal hypersensitivities, and risk increased with the number of modalities affected. Patients with interictal hypersensitivities also reported higher dizziness‐related disability, highlighting the need to systematically screen for these symptoms in clinical practice.

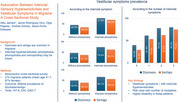

**Abbreviations**
DHI: Dizziness Handicap Inventory
GAD‐7: General Anxiety Disorder‐7
HIT‐6: Headache Impact Test
ICHD‐III: International Classification of Headache Disorders ‐III
Osmo: with osmophobia
Phono: with phonophobia
Photo: with photophobia
SPSS: Statistical Package for the Social Sciences
Wo osmo: without osmophobia
Wo phono: without phonophobia
Wo photo: without photophobia

## 1 Background

Although headache is considered the quintessential hallmark of migraine, approximately 51.7% of patients experience vestibular symptoms at some point (Vuković et al. 2007; Neuhauser and Lempert 2004). In fact, vestibular migraine is one of the most common causes of episodic vertigo (Dieterich et al. 2016). The clinical presentation of vestibular symptoms in migraine patients is highly varied and includes true vertigo, positional dizziness, or a sensation of disequilibrium (O'Connell Ferster et al. 2017). Even in the interictal phase, vestibular symptoms with increased levels of visual dependence have been identified (Agarwal et al. 2012).

Understanding this relationship is a complex issue, as vestibular symptoms may either be an intrinsic feature of migraine or due to a vestibular disorder. Hypotheses have been proposed linking peripheral mechanisms (vestibular sensitization) (Porta‐Etessam et al. 2011) and/or central mechanisms (dysfunction of vestibular sensory processing) (Kuritzky et al. 1981; Akdal et al. 2009; Casani et al. 2009; Cutrer and Baloh 1992). At the peripheral level, animal models of migraine suggest that sensitization of the trigemino‐vascular system, through its projections to the vestibular nucleus, may alter the sensitivity of these nuclei, leading to vestibular dysfunction (Zhang et al. 2020). The onset speed of the vestibular syndrome, its duration, symmetry, and degree of involvement determine the clinical manifestations, whether the patient presents with instability or true vertigo (Lucieer et al. 2016). At the central level, imaging studies have demonstrated significantly increased thalamic activation in patients with vestibular migraine during both vestibular stimulation (Russo et al. 2014) and migraine attacks (Shin et al. 2014). Furthermore, cutaneous allodynia, recognized as the principal clinical manifestation of central sensitization (Maleki et al. 2021), is notably more prevalent among patients with vestibular migraine (Toriyama et al. 2022).

Recent studies have shown that allodynia is linked to increased sensitivity in other sensory areas, resulting in symptoms like interictal photophobia (Cortez et al. 2021), phonophobia (Ashkenazi et al. 2010), and osmophobia (Lovati et al. 2015). However, there is limited research on the relationship between interictal sensory hypersensitivities and vestibular symptoms. To address this gap, we conducted a cross‐sectional study to investigate whether migraine patients with interictal sensory hypersensitivities experience higher prevalence and severity of dizziness and vertigo.

## 2 Methods

### 2.1 Participants

This cross‐sectional study used a survey to gather data from patients and their family members recruited from the Headache Unit of the Fundación Jiménez Díaz University Hospital in Madrid. The study protocol was approved by the Hospital Ethics Committee (PIC129‐23). Inclusion criteria included men or women, aged 18–65 years, with migraine according to the criteria of International Classification of Headache Disorders (ICHD)‐III (Headache Classification Committee of the International Headache Society (IHS) 2013). Participants were consecutively recruited from May 2023 to January 2024. No exclusions were made on the basis of comorbid vestibular or inner‐ear disorders. We did not adjudicate the etiology of vestibular symptoms; our primary aim was to capture their lifetime prevalence and burden within a real‐world migraine cohort.

### 2.2 Self‐Report Measures

All participants completed a self‐administered questionnaire, which included demographic variables and headache symptoms, including having ≥ 15 monthly headache days (MHDs), type of pain, topography, average intensity (on a scale of 1–10), exacerbation with head movements, nausea, and photophobia, phonophobia, and osmophobia (defined as discomfort or pain induced by light, sound, or odors), in order to establish a migraine diagnosis based on the criteria outlined in ICHD‐III (Headache Classification Committee of the International Headache Society (IHS) 2013). Because we did not collect all ICHD‐3 (Headache Classification Committee of the International Headache Society (IHS) 2013) elements required to classify chronic migraine (e.g., more than eight migraine days per month for more than 3 months), we did not assign episodic versus chronic migraine diagnostic labels. Instead, we treated ≥15 MHDs as a pragmatic proxy of disease chronicity for descriptive comparisons and as a covariate in regression models.

Moreover, participants were asked about any ongoing preventive treatments they were receiving and, if so, which one to prevent confusion with symptomatic treatments. The impact of the headaches was measured using the Spanish‐validated version of the Headache Impact Test (HIT‐6) scale (Martin et al. 2004). The questionnaire comprises six questions, each employing a scale with five ordered response categories, ranging from “*never*” to “*always*.” A total score is derived by summing the responses, with specific values assigned to each category: “*never*” = 6, “*rarely*” = 8, “*sometimes*” = 10, “*very often*” = 11, and “*always*” = 13. Consequently, the total scores can range from 36 to 78. Scores falling below 49 indicate little or no impact, while scores between 50 and 55 suggest some impact. Scores ranging from 56 to 59 indicate a substantial impact, while those at or above 59 denote severe impact. Additionally, symptoms indicative of central sensitization, such as interictal photophobia, phonophobia, and/or osmophobia, were assessed. These were defined as discomfort with light, noise, or odors occurring even in the absence of headache. Participants were asked the following question: “Do you have light sensitivity between migraine attacks?” An affirmative response classified the participant as having “migraine with interictal photophobia.” The same structure was used to assess interictal phonophobia and interictal osmophobia.

Participants were also asked about the presence of vestibular symptoms—specifically dizziness and vertigo—according to the definitions established by an international consensus committee of vestibular medicine specialists (Bisdorff et al. 2009). The following definitions were shown verbatim before each question: Vertigo was defined as “a sensation of self‐motion of the head or body when no actual motion is occurring, or a distorted perception of self‐motion during otherwise normal head movements.” Dizziness was defined as “a sensation of impaired or disturbed spatial orientation without a false or distorted sense of motion (e.g., giddiness, lightheadedness, nonspecific dizziness), excluding vertigo.” Participants then answered the following lifetime‐prevalence items: “Have you ever experienced vertigo as defined above?” (Yes/No) and “Have you ever experienced dizziness as defined above?” (Yes/No). For those who answered “Yes,” we recorded the chronology relative to migraine attacks using a single‐choice item: “When did this symptom typically occur in relation to your migraine attacks?” (Before/During/After/Independent of attacks). Additionally, the impact of these symptoms on daily life was evaluated using the Spanish‐validated version of the Dizziness Handicap Inventory (DHI) (Pérez et al. 2000). It comprises a 25‐item questionnaire assessing physical (seven questions, 28 points), functional (nine questions, 36 points), and emotional (nine questions, 36 points) factors associated with dizziness/unsteadiness. Total scores are categorized as no handicap (0–16 points), mild handicap (17–30 points), moderate handicap (31–60 points), or severe handicap (61–100 points).

Additionally, the Spanish‐validated version of the General Anxiety Disorder‐7 (GAD‐7) scale (García‐Campayo et al. 2010) was employed to evaluate the presence and severity of generalized anxiety. This questionnaire consists of seven questions assessing a 2‐week period. Scores range from 0 to 21, with 0–4 indicating minimal anxiety, 5–9 indicating mild anxiety, 10–14 indicating moderate anxiety, and scores greater than 15 indicating severe anxiety.

### 2.3 Statistical Analyses

No prior studies have examined differences in the prevalence of vestibular symptoms between migraine patients with and without interictal symptoms, making it challenging to calculate the sample size. However, the authors deemed a minimum difference of 12% between groups to be clinically meaningful. To ensure 80% statistical power and a 95% confidence level, a sample size of at least 270 participants was determined to be necessary. A descriptive analysis was conducted to outline demographic and baseline characteristics. Qualitative variables were summarized using frequencies and percentages, while quantitative variables were expressed as either mean and standard deviation (SD) or median and interquartile range (IQR), depending on their distribution, as assessed by the Kolmogorov–Smirnov test. Variables described with mean and SD were compared using the Student's *t*‐test, while those described with median and IQR were analyzed using the Mann–Whitney *U* test. The prevalence of vertigo and dizziness between groups with and without interictal sensitivities was assessed using odds ratios (OR) with 95% confidence intervals (95% CI). Differences in DHI scores between groups were analyzed using independent samples *t*‐tests, while symptom severity was evaluated with Pearson's chi‐square test. The prevalence and severity of vestibular symptoms by the number of interictal sensitivities were assessed using Pearson's chi‐square test, and DHI scores were analyzed with a one‐way ANOVA. Independent predictors associated with dizziness or vertigo were examined using a multivariable logistic regression analysis. First, a univariable logistic regression was performed to identify potential predictors. Variables with a *p *< 0.05 in the univariable analysis were evaluated for potential collinearity using the Variance Inflation Factor (VIF), particularly considering that interictal photophobia, interictal phonophobia, and interictal osmophobia may be highly related. Only variables without collinearity were included in the multivariable logistic regression model. The effect size and directionality of the estimates are reported as crude and adjusted ORs with 95% CIs. *p* values presented are for a two‐tailed test, with values < 0.05 considered statistically significant. We imputed variables with < 10% missing data using multiple imputation by chained equations (MICE), as this method is well‐suited for handling multiple types of variables and generating accurate imputations. The imputation process was configured to run for five iterations (*m* = 5), with a maximum of 50 iterations per imputation (maxit = 50), and a random seed of 500 to ensure reproducibility. Variables with missing data > 10% were not analyzed. Data analysis was performed using IBM SPSS Statistics software version 25 (IBM Corp., Armonk, NY, USA) and R version 4.4.0 (R Foundation for Statistical Computing, Vienna, Austria).

## 3 Results

**FIGURE 1:**
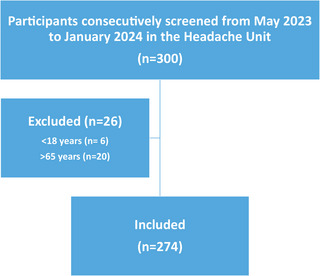
Flow chart of participant selection.

Of the 300 patients screened, a total of 274 with migraine were included. A total of 26 were excluded: 6 for being younger than 18 years and 20 for being older than 65 years (Figure 1). All participants met the ICHD‐3 criteria for migraine, except for criterion B (headache attacks lasting 4–72 h, untreated or unsuccessfully treated), which was not explicitly assessed. The mean age was 41.7 ± 12.4 years, with 239 females representing 87.2% of the sample. Regarding migraine characteristics, 83 participants (30.3%) experienced ≥ 15 MHDs, 165 (60.2%) reported unilateral pain, and 234 (85.4%) described throbbing pain. The mean pain intensity was 7.96 ± 1.5. Additionally, 239 participants (87.2%) reported headache exacerbation during physical activity, 193 (70.4%) experienced nausea, 250 (91.5%) reported photophobia, 249 (90.9%) experienced phonophobia, 166 (60.6%) had osmophobia, and 183 (67%) were on preventive treatment. The mean HIT‐6 score was 63.6 ± 7.0, with 78.5% of participants experiencing a severe impact. The GAD‐7 scale revealed that 62 (22.6%) exhibited minimal anxiety, 109 (39.8%) mild anxiety, 55 (20.1%) moderate anxiety, and 48 (17.5%) severe anxiety. Clinical characteristics are summarized in Table 1.

**TABLE 1:** Baseline characteristics, migraine features, interictal symptoms, impact of migraine (HIT‐6), and anxiety levels (GAD‐7) in the study sample.

Baseline characteristics	
*n* (%)	274 (70.6)
Age [years], median (SD)	41.7 (12.4)
Sex female, *n* (%)	239 (87.2)
**Migraine features**	
≥ 15 MHDs, *n* (%)	83 (30.3)
Unilateral pain, *n* (%)	165 (60.2)
Throbbing pain, *n* (%)	234 (85.4)
Intensity, median (SD)	7.96 (1.5)
Worsens with physical activity, *n* (%)	239 (87.2)
Nausea, *n* (%)	193 (70.4)
Photophobia, *n* (%)	250 (91.5)
Phonophobia, *n* (%)	249 (90.9)
Osmophobia, *n* (%)	166 (60.6)
On preventive treatment, *n* (%)	183 (67)

Interictal sensory hypersensitivities		
Type	Photophobia, *n* (%)	76 (27.7)
	Phonophobia, *n* (%)	67 (24.5)
	Osmophobia, *n* (%)	69 (25.2)
Number of sensory modalities	0, *n* (%)	143 (52.2)
	1, *n* (%)	70 (25.7)
	2, *n* (%)	41 (15)
	3, *n* (%)	20 (7.3)
**HIT‐6**		
Mean score, (SD)		63.7 (6.9)
Little or no impact, *n* (%)		13 (4.7)
Some impact, *n* (%)		20 (7.3)
Substantial impact, *n* (%)		26 (9.5)
Severe impact, *n* (%)		215 (78.5)
**GAD‐7**		
Mean (SD)		8.7 (5.2)
Minimal, *n* (%)		62 (22.6)
Mild, *n* (%)		109 (39.8)
Moderate, *n* (%)		55 (20.1)
Severe, *n* (%)		48 (17.5)

*Note*: No missing data were reported.

Abbreviations: GAD‐7, general anxiety disorder‐7; HIT‐6, Headache Impact Test; MHDs, monthly headache days.

### 3.1 Interictal Sensory Hypersensitivities

A notable portion of participants reported interictal sensory hypersensitivities: photophobia 76 (27.7%), phonophobia 67 (24.5%), and osmophobia 69 (25.2%). Among the participants, 143 (52.2%) did not report any interictal hypersensitivity symptoms, while 70 (25.7%) reported sensitivity in only one sensory modality, 41 (15%) in two modalities, and 20 (7.3%) in three modalities (Table 1).

### 3.2 Vestibular Symptoms

**FIGURE 2:**
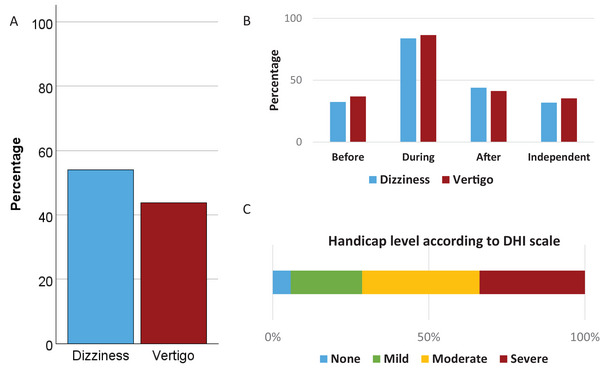
Vestibular symptom analysis in the total cohort. (A) Prevalence of dizziness and vertigo. (B) Chronological relationship between vestibular symptoms and migraine attacks. (C) The bar represents the distribution of disability caused by vestibular symptoms according to the DHI scale.

At least half of the patients exhibited vestibular symptoms, with 148 (54.0%) reporting dizziness, while 119 (43.8%) described experiencing vertigo (Table 2). These symptoms predominantly occurred during migraine attacks, with 83.8% of participants experiencing dizziness and 86.5% reporting vertigo during such episodes (Figure 2). The analysis of the DHI revealed a mean score of 50.2 ± 22.5. Among participants, 9 (6.1%) reported no handicap, 27 (18.2%) had a mild handicap, 59 (39.9%) had a moderate handicap, and 53 (35.8%) had a severe handicap (Figure 2).

**TABLE 2:** Vestibular symptom prevalence, chronological relationship with migraine attacks, and handicap in the total sample.

Vestibular symptoms	*n* = 274
Dizziness, *n* (%)	148 (54.0)
Before, *n* (%)	48 (32.4)
Missing	37 (25)
During, *n* (%)	124 (83.8)
Missing	11 (7.4)
After, *n* (%)	65 (43.9)
Missing	36 (24.3)
Independent, *n* (%)	47 (31.8)
Missing	34 (23.0)
Vertigo, *n* (%)	119 (43.8)
Before, *n* (%)	44 (36.9)
Missing	32 (26.9)
During, *n* (%)	103 (86.5)
Missing	11 (9.2)
After, *n* (%)	49 (41.2)
Missing	30 (25.2)
Independent, *n* (%)	42 (35.3)
Missing	31 (26.1)
DHI	
Score, mean (SD)	50.22 (22.5)
Handicap level	
None, *n* (%)	9 (6.1)
Mild, *n* (%)	27 (18.2)
Moderate, *n* (%)	59 (39.9)
Severe, *n* (%)	53 (35.8)

Abbreviation: DHI, Dizziness Handicap Inventory.

### 3.3 Vestibular Symptoms According to Interictal Sensorial Hypersensitivities

**FIGURE 3:**
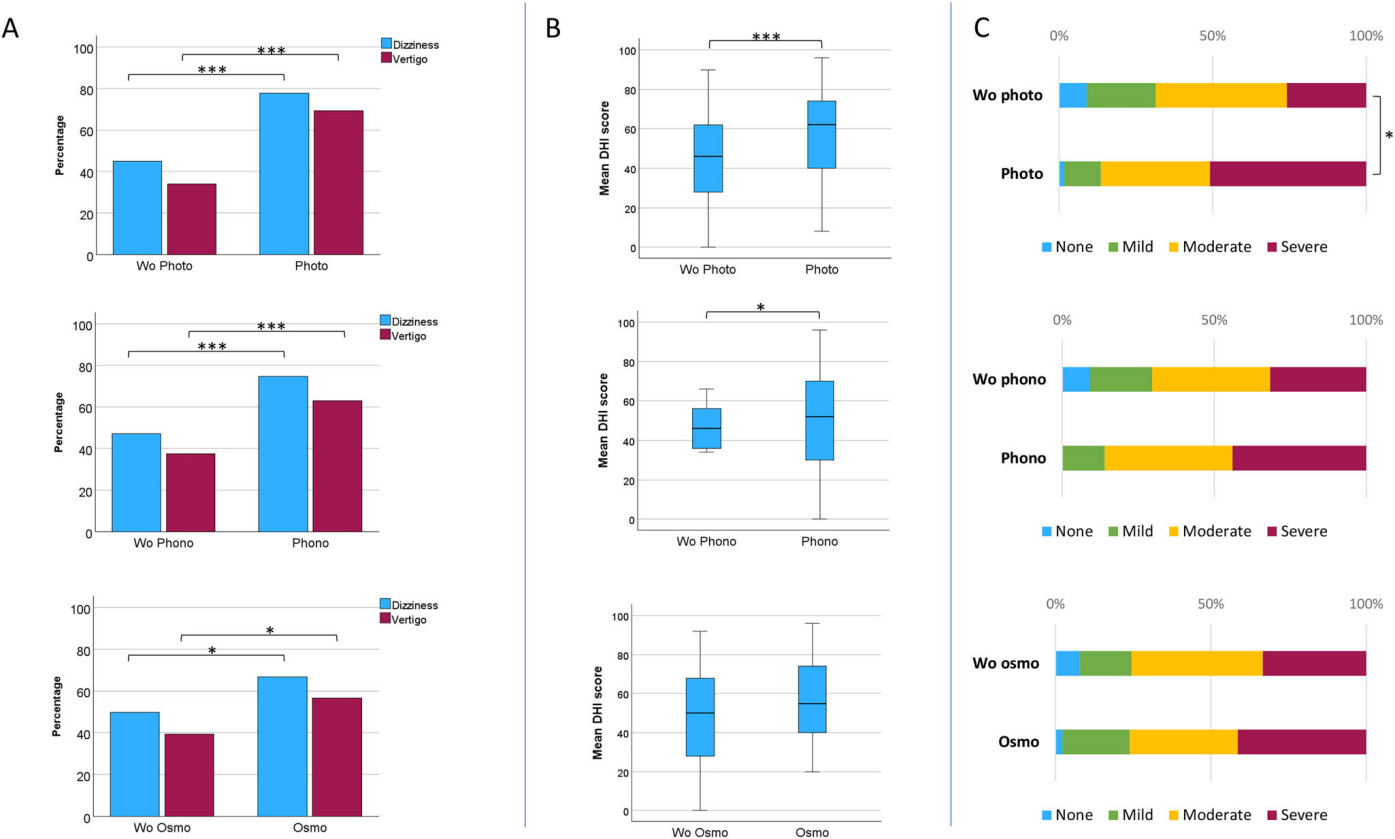
Vestibular symptoms by interictal sensory hypersensitivities. (A) Lifetime prevalence (%) of dizziness and vertigo in participants without versus with interictal photophobia (Wo photo vs. Photo), phonophobia (Wo phono vs. Phono), and osmophobia (Wo osmo vs. Osmo). Brackets indicate between‐group comparisons (Pearson's chi‐square). (B) Dizziness Handicap Inventory (DHI) total score among participants reporting vestibular symptoms (*n* = 148), stratified by the presence/absence of each interictal hypersensitivity. Box‐and‐whisker plots show the median (center line), interquartile range (box), and whiskers (range). (C) DHI disability levels (none, mild, moderate, severe) in the same subgroups, displayed as 100% stacked bars. *p < 0.05; **p < 0.01; ***p < 0.001.

**FIGURE 4:**
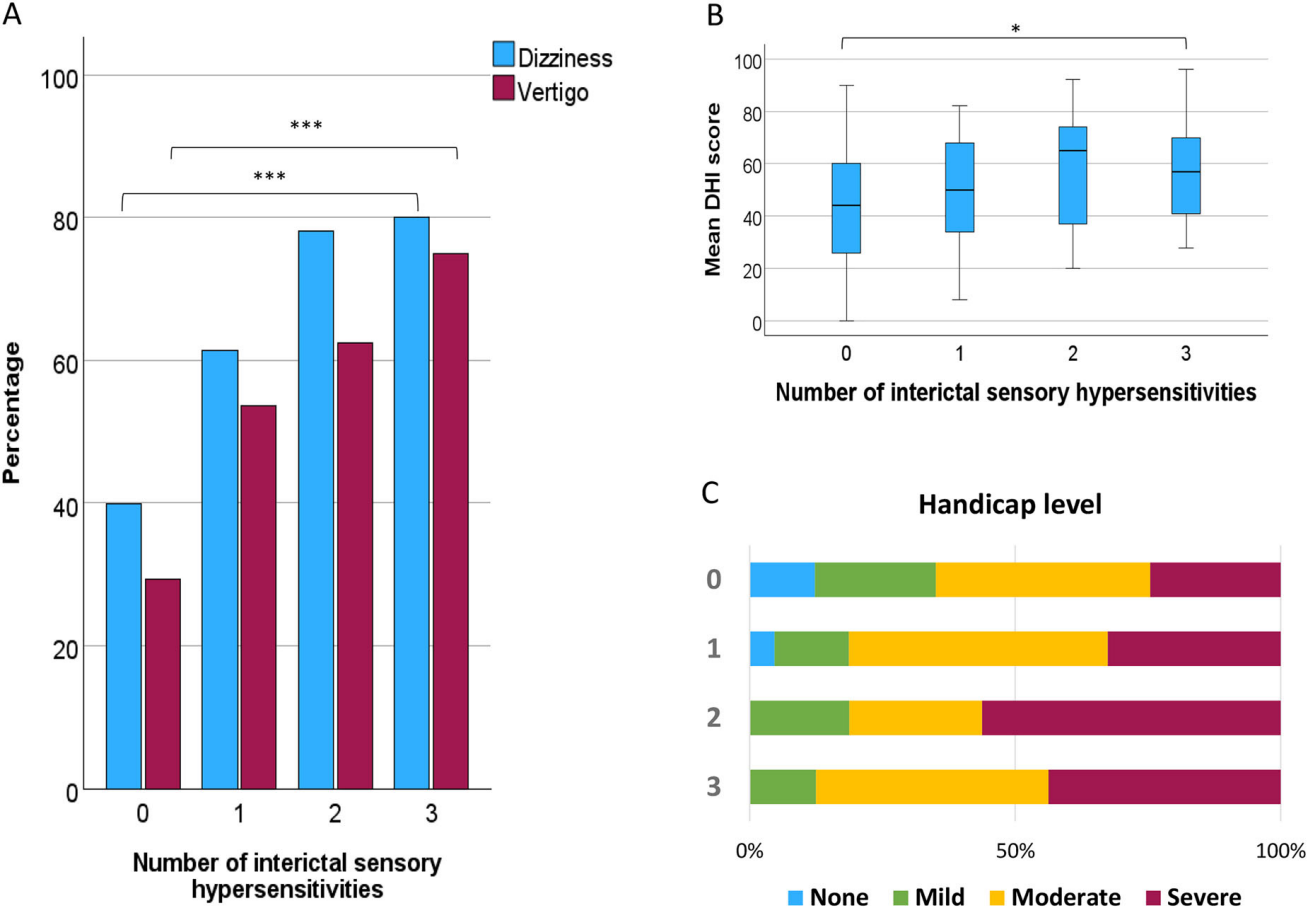
Analysis of vestibular symptoms based on the number of interictal sensory hypersensitivities. (A) Prevalence of dizziness and vertigo. (B) Vestibular symptom‐related handicap, with bars representing the mean score of the Dizziness Handicap Inventory (DHI) and error bars indicating the 95% confidence interval. (C) Bars illustrate the distribution of handicap severity. *p < 0.05; **p < 0.01; ***p < 0.001.

- Interictal photophobia:
  ○ Individuals with persistent photophobia exhibited significantly higher rates of dizziness (77.6% vs. 47.1% [OR 4.25; 95% CI, 2.31, 7.80; *p* < 0.001]) and vertigo (68.0% vs. 33.4% [OR 4.39; 95% CI: 2.47, 7.78; *p* < 0.001]) compared to those without it (Table 3).
  ○ Moreover, they also demonstrated higher DHI scale values (57.9 ± 20.7 vs. 45.1 ± 22.3, *p* = 0.001) and a greater percentage of patients with severe disability (50.8 vs. 25.8, respectively, *p* = 0.008) (Table 4).
- Interictal phonophobia:
  ○ Similarly, individuals with persistent phonophobia had a higher prevalence of dizziness (74.6% vs. 47.3% [OR 3.27; 95% CI: 1.77, 6.05; *p* < 0.001]) and vertigo (61.2% vs. 37.7% [OR 2.82; 95% CI: 1.59, 5.03; *p* < 0.001]) compared to those without it (Figure 3).
  ○ Additionally, the group showed higher DHI scale values (55.6 ± 20.2 vs. 47.5 ± 23.2, *p* = 0.037) and a greater percentage of subjects with severe disability (44.0 vs. 31.6, respectively, *p* = 0.090).
- Interictal osmophobia:
  ○ Likewise, individuals with persistent osmophobia had a higher prevalence of dizziness (66.7% vs. 49.8% [OR 2.02; 95% CI: 1.14, 3.57; *p* = 0.015]) and vertigo (56.5% vs. 39.4% [OR 2.00; 95% CI: 1.15, 3.47; *p* = 0.011]) compared to those without it.
  ○ Moreover, they exhibited higher DHI scale values (53.1 ± 22.3 vs. 48.9 ± 22.6, *p* = 0.299) and a greater percentage of individuals with severe disability (41.3 vs. 33.3, respectively, *p* = 0.374).
- Number of interictal sensory hypersensitive modalities:
  ○ The prevalence of dizziness increased progressively with the number of sensory sensitivity modalities. Among participants without any hypersensitivities, 39.9% experienced dizziness, which rose to 61.4% with one hypersensitivity, 78.0% with two, and 80.0% with three (*p* < 0.001). Similarly, the prevalence of vertigo followed a similar trend, increasing from 29.4% in participants without hypersensitivities to 52.8% with one, 60.9% with two, and 75.0% with three (*p* < 0.001) (Table 5).
  ○ The DHI scores also exhibited a gradual increase with the number of interictal hypersensitive modalities. The mean DHI scores were 43.5 ± 23.1 for participants without hypersensitivities, 50.6 ± 20.8 with one, 58.0 ± 22.2 with two, and 57.2 ± 19.8 with three (*p* = 0.014). Furthermore, the proportion of patients experiencing severe handicap escalated with the number of hypersensitivities, rising from 24.6% in participants without hypersensitivities to 43.8% in those with three (*p* = 0.050) (Figure 4).

**TABLE 3:** Prevalence of vestibular symptoms according to interictal sensory hypersensitivities.

Subgroup			Vestibular symptom prevalence					
Interictal symptoms		*n* = 274	Dizziness	OR (95% CI)	*p* ^a^	Vertigo	OR (95% CI)	*p* ^a^
Photophobia, *n* (%)	No	198 (72.3)	89 (47.1)	4.25 (2.31, 7.80)	**< 0.001**	67 (33.8)	4.39 (2.47, 7.78)	**< 0.001**
	Yes	76 (27.7)	59 (77.6)			52 (68.0)		
Phonophobia, *n* (%)	No	207 (75.5)	98 (47.3)	3.27 (1.77, 6.05)	**< 0.001**	78 (37.7)	2.82 (1.59, 5.03)	**< 0.001**
	Yes	67 (24.5)	50 (74.6)			41 (61.2)		
Osmophobia, *n* (%)	No	205 (74.8)	102 (49.8)	2.02 (1.14, 3.57)	**0.015**	80 (39.4)	2.00 (1.15, 3.47)	**0.011**
	Yes	69 (25.2)	46 (66.7)			39 (56.5		

^a^ Pearson's chi‐square test. Bold values indicate statiscally significant results (p < 0.05)

**TABLE 4:** Dizziness Handicap Inventory (DHI) in the 148 patients with vestibular symptoms according to interictal hypersensitivities.

Subgroup			Mean Score (SD)		Disability level				
Interictal symptoms		*n* = 148		*p* ^a^	None, *n* (%)	Mild, *n* (%)	Moderate, *n* (%)	Severe, *n* (%)	*p* ^a^
Photophobia, *n* (%)	No	89 (60.1)	45.1 (22.3)	< **0.001**	8 (9.0)	20 (22.5)	38 (42.7)	23 (25.8)	**0.008**
	Yes	59 (39.9)	57.9 (20.7)		1 (1.7)	7 (11.9)	21 (35.6)	30 (50.8)	
Phonophobia, *n* (%)	No	98 (66.2)	47.5 (23.2)	**0.037**	9 (9,2)	20 (20.4)	38 (38.8)	31 (31.6)	0.090
	Yes	50 (33.8)	55.6 (20.2)		0 (0.0)	7 (14.0)	21 (42.0)	22 (44.0)	
Osmophobia, *n* (%)	No	102 (68.9)	48.9 (22.6)	0.299	8 (7.8)	17 (16.7)	43 (42.2)	34 (33.3)	0.374
	Yes	46 (31.1)	53.1 (22.3)		1 (2.2)	10 (21.7)	16 (34.8)	19 (41.3)	

^a^ Independent samples *t*‐test; Pearson's chi‐square test. Bold values indicate statistically significant results (p < 0.05)

**TABLE 5:** Vestibular symptom prevalence and Dizziness Handicap Inventory scores according to the number of interictal sensory hypersensitivities.

Interictal hypersensitivities (*n* = 274)	0	1	2	3	*p* ^a^
*n*, (%)	143 (52.2)	70 (25.5)	41 (15.0)	20 (7.3)	
Vestibular symptom prevalence					
Dizziness, *n* (%)	57 (39.9)	43 (61.4)	32 (78.0)	16 (80.0)	**<** **0.001**
Vertigo, *n* (%)	42 (29.4)	37 (52.8)	25 (60.9)	15 (75.0)	**< 0.001**
Dizziness Handicap Inventory					
Mean score (SD)	43.5 (23.1)	50.6 (20.8)	58.0 (22.2)	57.2 (19.8)	**0.014**
None, *n* (%)	7 (12.3)	2 (4.7)	0 (0.0)	0 (0.0)	0.050
Mild, *n* (%)	13 (22.8)	6 (14.0)	6 (18.8)	2 (12.5)	
Moderate, *n* (%)	23 (40.4)	21 (48.8)	8 (25.0)	7 (43.8)	
Severe, *n* (%)	14 (24.6)	14 (32.6)	18 (56.3)	7 (43.8)	

^a^ Pearson's chi‐square test, ANOVA test. Bold values indicate statistically significant results (p < 0.05)

### 3.4 Multivariable Analyses

In the univariable analysis, most variables appeared to be associated with the presence of dizziness or vertigo. During the collinearity assessment, the number of interictal hypersensitivities, along with interictal photophobia, phonophobia, and osmophobia, was found to be collinear. This likely reflects their shared clinical construct, as these variables represent overlapping aspects of interictal sensory hypersensitivity. To address this, only the number of interictal hypersensitivities was included in the multivariable model to consolidate these effects into a single predictor, thereby avoiding redundancy and improving model stability (Table 6).

**TABLE 6:** Univariable and multivariable logistic regression analysis.

	Dizziness				Vertigo			
Variable	Univariable		Multivariable		Univariable		Multivariable	
	OR (95% CI)	*p*	OR (95% CI)	*p*	OR (95% CI)	*p*	OR (95% CI)	*p*
Interictal photophobia^a^	4.25 (2.36, 8.00)	**< 0.001**			4.39 (2.50, 7.89)	**< 0.001**		
Interictal phonophobia^a^	3.27 (1.80, 6.19)	**< 0.001**			2.83 (1.60, 5.09)	**< 0.001**		
Interictal osmophobia^a^	2.02 (1.15, 3.62)	**0.016**			2.00 (1.15, 3.50)	**0.014**		
Number of interictal hypersensitivities	2.10 (1.56, 2.83)	**< 0.001**	1.74 (1.24, 2.53)	**0.002**	2.02 (1.54, 2.69)	**< 0.001**	1.72 (1.24, 2.44)	**0.001**
Age	0.99 (0.97, 1.01)	0.323			1.00 (0.98, 1.02)	0.999		
Female sex	3.25 (1.53, 7.39)	**0.003**	2.99 (1.16, 8.17)	**0.027**	2.84 (1.29, 6.95)	**0.014**	2.51 (0.94, 7.36)	0.077
≥ 15 MHDs	3.39 (2.24, 7.14)	**< 0.001**	3.30 (1.68, 6.71)	**0.001**	3.55 (2.08, 6.17)	**< 0.001**	2.90 (1.51, 5.68)	**0.002**
Bilateral pain	2.00 (1.22, 3.31)	**0.006**	2.99 (1.61, 5.71)	**0.001**	1.89 (1.16, 3.11)	**0.011**	2.93 (1.57, 5.61)	**0.001**
Intensity	1.31 (1.11, 1.55)	**0.001**	0.87 (0.69, 1.10)	0.245	1.45 (1.22, 1.74)	**< 0.001**	0.95 (0.75, 1.21)	0.666
Worsens with physical activity	2.93 (1.43, 6.48)	**0.005**	2.99 (1.24, 7.68)	**0.018**	3.58 (1.59, 9.20)	**0.004**	3.29 (1.25, 9.76)	**0.022**
Throbbing pain	1.94 (0.99, 3.91)	0.057			2.30 (1.12, 5.01)	**0.028**	1.41 (0.58, 3.57)	0.457
Nausea	2.62 (1.54, 4.53)	**< 0.001**	2.84 (1.46, 5.66)	**0.002**	3.53 (1.99, 6.48)	**< 0.001**	3.86 (1.91, 8.18)	**< 0.001**
On preventive treatment	1.38 (0.83, 2.29)	0.216			1.36 (0.82, 2.29)	0.241		
HIT‐6	1.11 (1.07, 1.16)	**< 0.001**	1.08 (1.03, 1.14)	**0.005**	1.13 (1.08, 1.18)	**< 0.001**	1.08 (1.03, 1.15)	**0.006**
GAD‐7	1.12 (1.07, 1.18)	**< 0.001**	1.07 (1.01, 1.14)	**0.024**	1.10 (1.05, 1.16)	**< 0.001**	1.04 (0.98, 1.10)	0.196

Abbreviations: GAD‐7, general anxiety disorder‐7; HIT‐6, Headache Impact Test; MHDs, monthly headache days.

^a^ To address collinearity, the variables interictal photophobia, interictal phonophobia, and interictal osmophobia were excluded, and only the number of interictal hypersensitivities was included in the multivariable analysis. Bold values indicate statistically significant rsults (p < 0.05)

In the multivariable logistic regression analysis, the number of interictal hypersensitivities was significantly associated with both dizziness (OR: 1.74 [95% CI: 1.24, 2.53; *p* = 0.002]) and vertigo (OR: 1.72 [95% CI: 1.24, 2.44; *p* = 0.001]), indicating that each additional hypersensitivity increased the likelihood of these symptoms. Female sex was associated with nearly three times the risk of dizziness compared to males (OR: 2.99 [95% CI: 1.16, 8.17; *p* = 0.027]). Additionally, having ≥ 15 MHDs significantly increased the risk of both dizziness (OR: 3.30 [95% CI: 1.68, 6.71; *p* = 0.001]) and vertigo (OR: 2.90 [95% CI: 1.51, 5.68; *p* = 0.002]). Bilateral pain was associated with dizziness (OR: 2.99 [95% CI: 1.61, 5.71; *p* = 0.001]) and vertigo (OR: 2.93 [95% CI: 1.57, 5.61; *p* = 0.001]). Similarly, movement sensitivity showed significant associations with dizziness (OR: 2.99 [95% CI: 1.24, 7.68; *p* = 0.018]) and vertigo (OR: 3.29 [95% CI: 1.25, 9.76; *p* = 0.022]). Nausea emerged as a strong predictor of both dizziness (OR: 2.84 [95% CI: 1.46, 5.66; *p* = 0.002]) and vertigo (OR: 3.86 [95% CI: 1.91, 8.18; *p* < 0.001]). Furthermore, higher scores on the HIT‐6 scale were significant predictors of dizziness (OR: 1.08 [95% CI: 1.03, 1.14; *p* = 0.005]) and vertigo (OR: 1.08 [95% CI: 1.03, 1.15; *p* = 0.006]). In contrast, the GAD‐7 score was associated only with dizziness (OR: 1.07 [95% CI: 1.01, 1.14; *p* = 0.024]). Finally, pain intensity and throbbing pain were not significantly associated with vestibular symptoms in the final model (Table 6).

## 4 Discussion

The results presented in this paper provide insights into the relationship between dizziness, vertigo, and interictal sensory hypersensitivities among migraine patients. Consistent with previous research, our findings confirm that a substantial proportion of migraine patients experience vestibular symptoms (Vuković et al. 2007; Neuhauser and Lempert 2004), particularly during migraine attacks (Lampl et al. 2019), leading to severe handicap in a significant subset of this population (Balcı and Akdal 2020).

In our study, the prevalence of dizziness was 54.0%, and that of vertigo was 43.8%, consistent with previous reports indicating that between 30% and 50% of patients with migraine experience vestibular symptoms—such as vertigo, dizziness, or balance disturbances—at some point during the course of their condition (Carvalho et al. 2019). This concordance highlights the high frequency of vestibular manifestations in migraine and underscores their clinical relevance in the comprehensive assessment of these patients.

Nearly one‐third of migraine patients exhibited some form of interictal sensory hypersensitivity. Among patients with interictal photophobia, the risk of experiencing dizziness or vertigo was four times higher, approximately three times higher for those with interictal phonophobia, and around twice as high for those with interictal osmophobia. Additionally, this group of patients demonstrated higher levels of disability attributable to vestibular symptoms, as reflected by higher DHI scores, with a greater proportion experiencing severe disability. However, statistical significance was observed only in the groups with interictal photophobia and phonophobia.

Moreover, our study revealed that for each additional hypersensitive sensory modality, the risk of experiencing dizziness or vertigo increased by 70%. A greater number of interictal sensory hypersensitivities was also associated with a higher degree of disability. Previous studies have shown that as the number of hypersensitivity symptoms increases, so does the degree of disability related to headache (Suzuki et al. 2021). Therefore, individuals with migraine who also experience hypersensitivity in multiple sensory modalities have greater disability not only due to headache itself but also due to vestibular symptoms.

Consistent with previous studies, the multivariable analysis demonstrated that a higher burden of migraine, evidenced by ≥ 15 MHDs, was a strong predictor of dizziness and vertigo (Lampl et al. 2019). Women were found to have three times the risk of experiencing dizziness, possibly due to the widespread distribution of estrogen receptors in vestibular processing regions, including the hypothalamus, cerebellum, and brainstem. Fluctuations in estrogen levels—particularly during menstruation, pregnancy, or menopause—have been suggested to exacerbate not only migraines but also dizziness and vertigo (Villar‐Martinez and Goadsby 2024).

Participants with bilateral pain had a threefold higher risk of vestibular symptoms, which may be explained by the sensitization of third‐order neurons linked to the development of cutaneous allodynia on the contralateral side of the head (Burstein et al. 2000). Allodynia is notably more prevalent among patients with vestibular migraine (Toriyama et al. 2022), suggesting a connection between bilateral pain and vestibular symptoms. However, this relationship could not be confirmed in our study, as allodynia was not specifically assessed.

Movement sensitivity during migraine attacks is attributed to the sensitization of peripheral nociceptors innervating intracranial blood vessels and the meninges (Strassman et al. 1996). Given that this symptom tripled the risk of dizziness and vertigo, a potential link between central and peripheral sensitization in the development of these symptoms may exist. Patients with nausea had nearly three times the risk of experiencing dizziness and almost four times the risk of experiencing vertigo. The mechanism underlying nausea in migraine patients likely involves hypothalamic dysfunction and dopamine hypersensitivity (Goadsby et al. 2017). Previous studies have examined the convergence of vestibular and autonomic afferent pathways (Balaban 1999). Our findings suggest that the co‐occurrence of nausea and vestibular symptoms may be linked to an overlap in their underlying mechanisms. Finally, although previous studies have associated anxiety with vestibular symptoms (Kim et al. 2023), in our study, it was only linked to a slight increase in the risk of dizziness but not vertigo.

Ongoing preventive therapy could theoretically mitigate interictal sensory hypersensitivities and vestibular manifestations. Although preventive status was not significantly associated with dizziness or vertigo in univariable analyses and was not included in multivariable models per our prespecified approach, residual confounding cannot be excluded. Moreover, heterogeneity across preventive classes, dosing, and treatment duration was not analyzed, and preventive status was captured cross‐sectionally, whereas vestibular outcomes were assessed as lifetime prevalence. These design features would be expected to bias associations toward the null rather than generate spurious positives.

Regarding the relationship between vestibular symptoms and sensitization, evidence suggests that this phenomenon can occur at various levels. According to a study, vestibular symptoms may arise from sensitization of the vestibular nuclei, which are influenced by migraine‐related brainstem regions and simultaneously regulated by inhibitory feedback from the cerebellar nodulus and uvula, where canal‐otolith integration occurs (King et al. 2019).

Furthermore, the thalamus serves as a relay center for processing incoming sensory information, including the vestibular network (Kirsch et al. 2016). It also processes dural nociceptive inputs through higher‐order neurons located in the posterior, lateral posterior, and lateral dorsal nuclei (Noseda et al. 2011). These nuclei subsequently project to various cortical regions, such as the auditory, visual, and olfactory cortices and parietal association cortices, among others, to manage the processing of vestibular sensory information (Noseda et al. 2011). Imaging studies have indicated significantly increased thalamic activation in patients with vestibular migraine during both vestibular stimulation (Russo et al. 2014) and migraine attacks (Shin et al. 2014). This suggests that thalamic sensitization due to repeated migraine episodes may not only cause distortion of cutaneous sensation (allodynia) but also disrupt spatial perception, potentially leading to vertigo and/or dizziness.

Finally, the cerebral cortex is hyperexcitable in individuals with migraine (Denuelle et al. 2011), a phenomenon possibly mediated by brainstem nuclei like the locus coeruleus and dorsal raphe (Filippov et al. 2008; Filippov et al. 2004; Devilbiss et al. 2006). Certain studies have noted that vestibular migraine is linked to abnormal cortical interactions between visual and vestibular networks (Shin et al. 2014; Bednarczuk et al. 2019). Furthermore, there is evidence of reduced motion perception thresholds and heightened susceptibility to motion sickness among patients with vestibular migraine during interictal periods, suggesting disruptions in higher‐order vestibular processing (Wurthmann et al. 2021).

Our results have several implications for clinical practice. First, they support brief, routine screening for interictal sensory hypersensitivities (photophobia, phonophobia, and osmophobia) and vestibular symptoms in patients with migraine, given their added disability. When present, the DHI provides a rapid estimate of functional impact and can help prioritize referrals. Because the number of interictal hypersensitivities was associated with dizziness and/or vertigo, these features may be used for simple risk stratification to identify patients who could benefit from closer follow‐up or targeted interventions. This is particularly relevant because, in our experience, many patients do not spontaneously link these symptoms to migraine and therefore do not report them unless specifically asked. Although robust evidence for pharmacologic treatments aimed specifically at interictal hypersensitivities is currently limited, patients with prominent vestibular complaints or elevated DHI scores may benefit from vestibular rehabilitation and graded habituation strategies.

Several limitations of this study must be acknowledged. Firstly, all data were collected through a self‐administered questionnaire, which may introduce recall bias. Although the diagnosis of migraine was corroborated based on patient‐reported symptoms, criteria A and B of the ICHD‐3 were not explicitly evaluated. Additionally, our analysis of dizziness and vertigo was based on their lifetime prevalence as symptoms rather than as diagnostic entities, which limits the specificity of our findings. While most participants reported vestibular symptoms during migraine attacks, we did not exclude individuals with other vestibular or neurological disorders and did not adjudicate etiology. These symptoms could therefore reflect other conditions—such as persistent postural‐perceptual dizziness (Sarna et al. 2021), which is highly prevalent in migraine—or other vestibular syndromes associated with migraine. Vertigo is also recognized as a migraine trigger, meaning the two may coexist as independent conditions that appear temporally related. The cross‐sectional design of this study further restricts our ability to establish causal relationships between migraine, interictal hypersensitivities, and vestibular symptoms. Moreover, the reliance on a single time point prevents us from capturing potential temporal fluctuations in symptom severity or frequency. Furthermore, we did not assess cutaneous allodynia, a recognized clinical marker of central sensitization that has been associated with a higher prevalence of vestibular symptoms (Toriyama et al. 2024); its omission precludes us from evaluating its potential role in the observed associations. In addition, in some patients, vestibular symptoms may represent a form of migraine aura, although our study design did not allow us to differentiate these from symptoms occurring in other contexts. Finally, dizziness can also be an adverse effect of acute and preventive migraine medications. While we recorded ongoing preventive therapy, we did not collect detailed data on lifetime drug exposure or the temporal relationship between treatment initiation and vestibular symptoms. These factors could have influenced our findings and warrant consideration in future studies.

This study has several notable strengths. First, it provides valuable insights into the relationship between vestibular symptoms, interictal sensory hypersensitivities, and migraine, an area that remains underexplored. The inclusion of a relatively large sample size enhances the robustness of our findings and allows for meaningful subgroup analyses. The use of standardized tools, such as the DHI, ensures the objective measurement of vestibular symptom burden. Regarding the generalizability of the findings, the study was conducted exclusively within medical settings, encompassing patients with a significant migraine burden, the majority of whom required preventive treatment (67%). Consequently, cases where individuals did not seek care from a physician are underrepresented.

## 5 Conclusions

Migraine patients with interictal photophobia, phonophobia, and osmophobia are at a higher risk of experiencing dizziness and vertigo, with the risk and severity increasing proportionally to the number of hypersensitive sensory modalities. Factors such as having ≥ 15 MHDs emerged as a strong predictor of dizziness, while nausea was significantly associated with vertigo. These findings are not only relevant from a pathophysiological perspective but also hold significant clinical implications, as patients with interictal sensory hypersensitivities exhibit a greater degree of disability, as evidenced by elevated DHI scores.

## Author Contributions

**Alex Jaimes**: conceptualization, investigation, writing – original draft, methodology, validation; software, formal analysis, project administration, data curation, visualization. **Jaime Rodríguez‐Vico**: writing – review and editing, data curation, validation, resources. **Andrea Gómez**: writing – review and editing, validation, data curation, resources. **Olga Pajares**: writing – review and editing, validation, data curation, resources. **Jesús Porta‐Etessam**: investigation, writing – review and editing, supervision.

## Ethics Statement

The study obtained approval from the Research Ethics Committee of the Fundación Jiménez Díaz University Hospital (protocol number PIC129‐23_FJD). All participants were provided with detailed information about the study, and informed digital consent was obtained.

## Conflicts of Interest

Alex Jaimes has received honoraria from Lilly, TEVA, and Allergan‐AbbVie. Jaime Rodríguez‐Vico has received honoraria from Lilly, TEVA, Novartis, Allergan‐AbbVie, and Exeltis, and research support from Allergan‐Abbie. The other authors declare no conflicts of interest.

## Peer Review

The peer review history for this article is available at https://publons.com/publon/10.1002/brb3.70874.

## Data Availability

Data are available from the corresponding authors on request.
